# Quantitative Compositional Mapping on a Micrometer Scale

**DOI:** 10.6028/jres.093.141

**Published:** 1988-06-01

**Authors:** Dale E. Newbury

**Affiliations:** Microanalysis Research Group, Center for Analytical Chemistry, National Bureau of Standards, Gaithersburg, MD 20899

## Introduction

Quantitative electron microprobe analysis has been restricted traditionally to application at single points or along scan vectors generated by beam or stage scans. However, from the very beginning of this field, the direct visualization of elemental spatial distributions by means of x-ray area scans or “dot maps” has been a powerful adjunct to quantitative analysis [1]. A dot map is created by marking the beam positions at which characteristic x-rays are detected by writing a white dot on a cathode ray tube scanned in synchronism with the beam position on the specimen. The spatial distribution of a selected elemental constituent can be depicted with a dot map at the micrometer level of resolution. Because of the great inherent value of information presented in a visual fashion, dot maps have become a major operating mode of the electron microprobe. However, dot maps are qualitative in nature and suffer from several key deficiencies. Quantitative information is lost because count rates are not recorded; the image information is recorded on film and is inflexible for subsequent processing; the ability to detect mass concentrations below 10 weight percent is severely limited by time constraints; and the method suffers from poor sensitivity to small concentrations changes measured against a high average concentration level.

## Compositional Mapping

The development of true compositional mapping with the electron microprobe has recently been achieved in the microprobe laboratories at the National Bureau of Standards working in conjunction with the National Institutes of Health [2]. The basic methodology consists of recording under computer control scan matrix arrays of actual x-ray count rates, derived from both wavelength-dispersive and energy-dispersive x-ray spectrometers. These x-ray intensity matrices are then subjected, on a pixel-by-pixel basis, to complete conventional quantitative analysis procedures. These procedures include all of the usual steps, such as deadtime correction, background correction, standardization against intensities measured on pure elements or simple compounds, and matrix factor correction to account for effects due to electron scattering, x-ray absorption, and secondary fluorescence [3]. In addition to these conventional corrections, specific corrections must be applied to scanned images to compensate for the effect of defocussing of the wavelength-dispersive spectrometers which arises when the beam is scanned off the optic axis of the instrument. This defocus correction can be applied either mechanically by scanning the specimen stage or by rocking the diffraction crystal in synchronism with the scan. Alternatively, the correction can be generated mathematically by making use of the geometric equivalence between spatial scanning on the specimen with a fixed spectrometer diffraction condition and scanning the x-ray peak with the spectrometer while the beam is fixed on the specimen. This equivalence can be used to calculate the spatial dependence of the defocussing at any magnification from a spectrometer scan of the peak [4].

**Figure 1:**
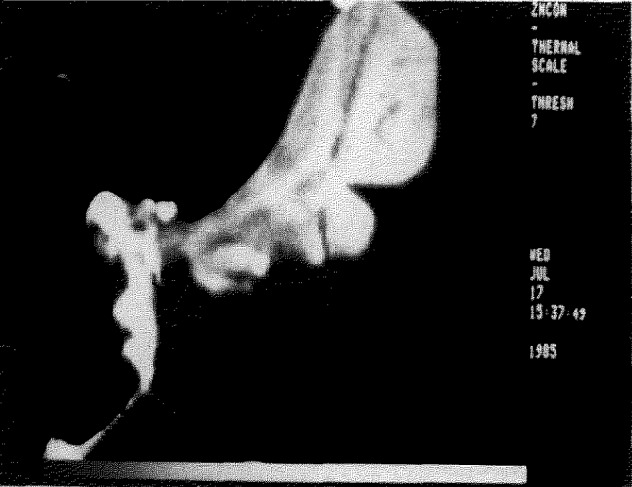
Quantitative compositional map of zinc at the grain boundaries of polycrystalline copper. Concentrations ranging from 0–10 weight precent are mapped. Image field width = 100 micrometers.

**Figure 2:**
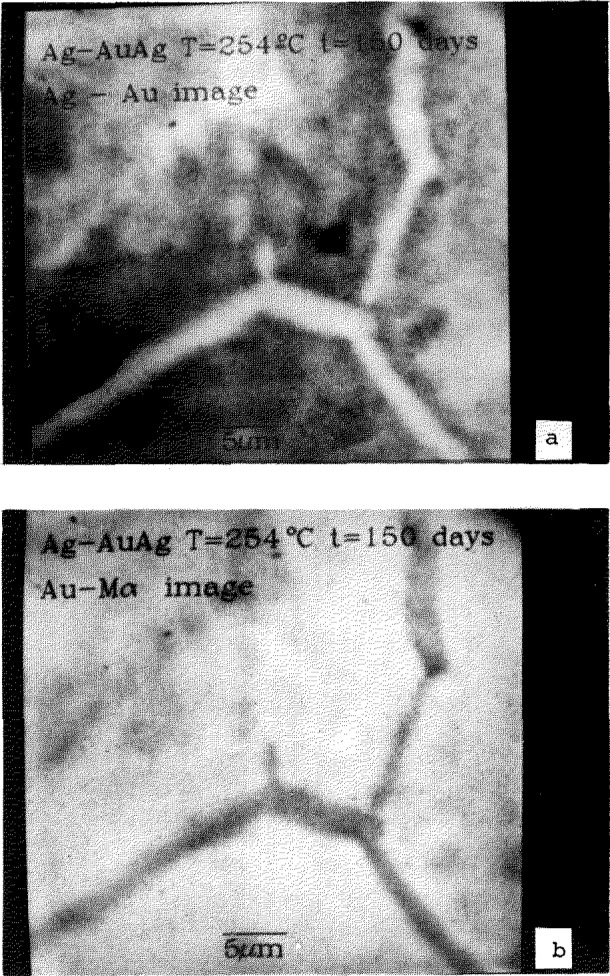
Quantitative compositional maps of grain boundary regions in polycrystalline silver-gold alloy: a) distribution of silver, with contrast enhancement to show an enrichment of 5 weight percent silver against a background of 65 weight percent silver; b) corresponding gold image, showing the deficiency of gold in the grain boundary region. Image field width = 50 micrometers.

The resulting matrices of concentration values can be displayed as images in which the gray level or color encoding is based not merely upon the x-ray intensities but rather upon the actual concentrations of the elemental constituents. An example of a quantitative compositional map of a zinc diffusion zone at the grain boundaries of a polycrystalline copper sample is shown in figure 1 [5]. This image, which was collected in a matrix scan of 9 hours duration, depicts concentrations as low as 0.2 weight percent. The digital compositional images can be subjected to a wide variety of image processing algorithms to enhance the visibility of weak contrast and to highlight specific composition ranges, substantially improving the flow of information and overcoming many of the limitations of the classical dot mapping method. For example, as shown in figure 2a, by making use of digital processing to achieve contrast expansion, it becomes possible to view small concentration changes against a high average background, e.g., a 5 weight percent change in the silver constituent can be seen against a general background of 65 weight percent silver for grain boundary diffusion zones in a polycrystalline silver-gold alloy. The corresponding deficiency in the gold constituent in this specimen can also be visualized, as shown in figure 2b. In addition to various gray scale image processing algorithms, color scales can also be employed with digital image processing to improve the visibility of desired information. Through the use of carefully constructed “logical” color scales, such as the thermal color sequence (which involves assigning a concentration scale to the color sequence: black, red, orange, yellow, white), compositional ranges covering two decades can be directly visualized. Most importantly, the compositional images are supported by the availability of the quantitative compositional information, including statistics, at every pixel. This information is available for interrogation at any time. Thus, when a structure of interest is recognized in the compositional image, the actual concentrations determined at individual pixels, vectors or areas can be immediately examined.

The extension of quantitative compositional mapping to reach “trace” levels of detection in images depends on two requirements. First, the analytical system must be capable of performing with instrumental stability in electron dose and x-ray detection efficiency to less than 0.1 percent deviations over the long time periods, of the order of 10 hours, which are required to accumulate sufficient x-ray counts. Secondly, since the x-ray bremsstrahlung is strongly dependent upon atomic number, accurate modeling of the x-ray background is needed in order to subtract this contribution from the peak intensities [6]. With careful attention paid to the problem of background correction, compositional mapping has been demonstrated to permit imaging of concentration levels as low as 1000 ppm in materials of intermediate and high atomic number, and levels of 100 ppm can be detected in low atomic number systems such as biological specimens [7].

While this new methodology will not replace conventional single point analysis because of the time requirements of compositional imaging, the combination of compositional images and quantitative analysis is a powerful aid to the solution of many problems. The advent of increasingly powerful laboratory computer systems will allow implementation of imaging systems as a common feature in future analytical facilities.
